# Study of active neighborhoods in Detroit (StAND): study protocol for a natural experiment evaluating the health benefits of ecological restoration of parks

**DOI:** 10.1186/s12889-020-08716-3

**Published:** 2020-05-07

**Authors:** Amber L. Pearson, Karin A. Pfeiffer, Joseph Gardiner, Teresa Horton, Rachel T. Buxton, Ruth F. Hunter, Victoria Breeze, Thomas McDade

**Affiliations:** 1 Department of Geography, Environment and Spatial Sciences, Michigan State University, 673 Auditorium Road, East Lansing, MI 48824 USA; 2 Department of Kinesiology, Michigan State University, 27R Intramural Rec Sports- Circle, 308 West Circle Drive, East Lansing, MI 48824 USA; 3 Department of Epidemiology and Biostatistics, Michigan State University, 909 Wilson Road, Room B601, East Lansing, MI 48824 USA; 4 Department of Anthropology, Northwestern University, 1810 Hinman Ave, Evanston, IL 60208 USA; 5 Department of Biology, Carleton University, 209 Nesbitt Biology Building, Ottawa, ON K1S 5B6 Canada; 6 Centre for Public Health, Queen’s University Belfast, University Road, Belfast, BT7 1NN Northern Ireland, UK

**Keywords:** Low-income, Built environment, Greenspace, Urban planning, Physical activity, Stress, Green exercise

## Abstract

**Background:**

Individuals living in deprived inner cities have disproportionately high rates of cancers, Type 2 diabetes and obesity, which have stress- and physical inactivity-related etiologies. This study aims to quantify effects of ecological park restoration on physical activity, stress and cardio-metabolic health outcomes.

**Methods:**

The Study of Active Neighborhoods in Detroit is a quasi-experimental, longitudinal panel natural experiment with two conditions (restored park intervention (INT) and control (CNT)) and annual measurements at baseline and 3-years post-restoration. Individuals (sampled within 500 m of an INT/CNT park) serve as the unit of analysis. Restoration (*n* = 4 parks) involves replacing non-native plants and turf with native plants; creating trails; posting signage; and leading community stewardship events. The CNT condition (*n* = 5) is an unmaintained park, matched to INT based on specified neighborhood conditions. Recruitment involves several avenues, with a retention goal of 450 participants. Park measures include plant/avian diversity; usage of the park (SOPARC); signs of care; auditory environment recordings; and visual greenness using 360 imagery. Health outcomes include device-based physical activity behavior (primary outcome); salivary cortisol (secondary outcome); and several downstream health outcomes. Exposure to the INT will be assessed through visual contact time and time spent in the park using GPS data. Changes in health outcomes between years and INT versus CNT will be tested using generalized linear (mixed) models.

**Discussion:**

Our study will examine whether restored urban greenspaces increase physical activity and lower stress, with public health planning implications, where small changes in neighborhood greenspaces may have large health benefits in low-income neighborhoods.

**Study Registration:**

Registration: OSF Preregistration registered March 31, 2020. Accessible from https://osf.io/surx7.

## Background

Individuals living in socioeconomically deprived inner cities have disproportionately high rates of obesity, Type 2 diabetes, cancer and cardio-metabolic conditions, all of which have stress- and physical inactivity-related etiologies [1–14]. The cost of these diseases is enormous, where the direct annual medical costs for obesity exceed $300b [15], and societal tolls include declining or stagnating life expectancy, particularly in low-income communities [16]. Low-income neighborhoods experience dual risks, whereby physical activity (PA) levels are low and stress levels are high [17, 18]. Stress and physical inactivity can both lead to downstream inflammatory changes that alter body composition and metabolic and immune functions linked to chronic disease [19–22]. Studies underscore the twinned benefits of weight management and lowered stress that engaging in PA confers [23]. Lower stress, regardless of PA, assists with sustained weight loss [24] and improved cardio-metabolic health [25], making it an attractive goal for health benefits.

To induce change in PA and stress on a population level, researchers and city planners are exploring features of the built environment, such as greenspace (e.g., parks), that may promote healthy lifestyles [26, 27]. Parks serve as places to engage in PA in direct contact with nature — called ‘green PA’ — which has been shown to lower anxiety [28] and perceived stress [29, 30] over and above the effects of indoor PA [23] or outdoor PA without greenery [31]. In addition, cross-sectional research indicates that passive exposure to greenspace (e.g., visual, as in the sight of plants and trees, and auditory, as in birdsong) may lower stress [32, 33]. However, not all residents living near parks visit the parks or engage in PA, and, empirically, the relationship between neighborhood greenspace and PA is inconsistent (for a review [34]). Even so, greener neighborhoods consistently predict lower obesity rates across age groups and rural/urban settings [35–41]. One possible explanation for these findings is that healthier people simply choose to live in greener areas. Yet another possible explanation is that the greenness-obesity relationship is influenced not only by PA but by stress reduction.

To address these knowledge gaps, we designed the Study of Active Neighborhoods Detroit (StAND) to utilize a natural experiment (not a behavioral intervention) to illuminate the causal health effects of greenspace. We integrate leading-edge geospatial techniques to assess individual-level exposure to greenspace with longitudinal evaluation of device-based measures of PA and biomarkers of stress and cardio-metabolic health in low-income, predominantly African American individuals living in neighborhoods in Detroit, Michigan U.S.A.

### Objectives

The aims of StAND are to observe the effects of ecological restoration of parks on PA, stress and cardio-metabolic health outcomes from baseline through three-years post restoration using a quasi-experimental design. The hypotheses are:

#### Hypothesis 1

Compared to participants in control (CNT) park neighborhoods, participants in intervention (INT) park neighborhoods will have increased PA levels at three-years post-restoration.

#### Hypothesis 2

Compared to participants in CNT park neighborhoods, participants in INT park neighborhoods will have increased levels of ‘green PA’ at three-years post-restoration.

#### Hypothesis 3

Across both INT and CNT parks, the quality of visual and auditory exposures (positive and negative) will affect PA levels.

#### Hypothesis 4

Compared to participants in CNT park neighborhoods, participants in INT park neighborhoods will have lower stress levels as indexed by measures of cortisol, perceived stress, and anxiety at three-years post-restoration.

#### Hypothesis 5

Participants with higher levels of PA and ‘green PA’ will have lower stress.

#### Hypothesis 6

The quality of visual and auditory exposures (positive and negative) will affect stress levels.

#### Hypothesis 7

Compared to participants in CNT park neighborhoods, participants in INT park neighborhoods will have improved glycated hemoglobin A1C (A1C) and C-reactive protein (CRP) at three-years post-restoration.

#### Hypothesis 8

Compared to participants in CNT park neighborhoods, participants in INT park neighborhoods will have lower blood pressure, body mass index (BMI), and hip-to-waist ratios at three-years post-restoration.

## Methods

### Design

**Fig. 1:**
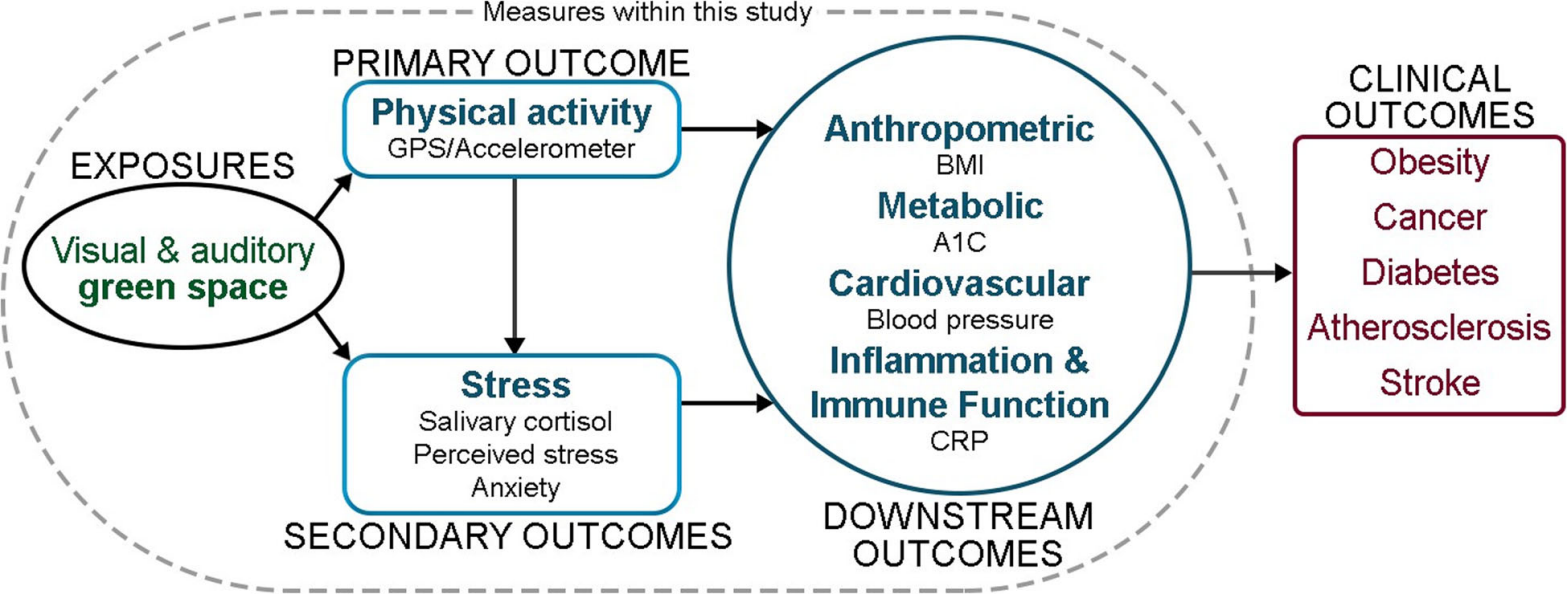
Study of active neighborhoods in Detroit (StAND) conceptual diagram of study exposures and outcomes. Items enclosed in the dotted oval will be measured in this study

The overall design of StAND is a four-year quasi-experimental, natural experiment, with two conditions (INT and CNT), four measurement occasions, and individual-level measurement of exposures and outcomes (see Fig. 1 for a conceptual diagram for the study). Four INT parks were selected by Detroit Audubon and five comparable CNT parks were selected, based on size, existing trees/plants, and proximity to other bird habitats. All adults (i.e. aged ≥18 years) living in a CNT or INT park neighborhood (defined in the following text) were invited to participate. Baseline (t = 0) and three annual measurements (t = 1 to t = 3) post-restoration will be conducted on participating individuals with the intention of having a longitudinal panel study design. However, we considered sampling alternatives depending on attrition, as others studies have done [42]. If attrition is higher than expected (> 25% at t = 1), then additional participants will be recruited at each time point, yielding a repeated cross-sectional design, with a nested panel. If assessments at certain time points are missing within the panel for some participants, we will assume that data from our unbalanced panel is missing at random.

The study was approved by the Michigan State University’s Institutional Review Board (IRB Approval #STUDY00000587; date 03/21/2019). The study was registered with OFS (osf.io/surx7) and successfully tested in a pilot study (Pearson AL, Clevenger K, Horton TH, Gardiner J, Asana V, Dougherty B, et al: Feelings of safety during daytime walking: Associations with mental health, physicial activity and cardiometabolic health in two high vacancy, low-income neighborhoods in Detroit, Michigan, in review) [43]. The protocol was completed using the TREND guidelines [44].

### Participants

Participants are adults who reside in a study park’s zone of influence, which is deemed to be 0.5 km (0.31mi), which is an approximate 15-min walk to the park and also a reasonable maximum distance for hearing birdsong in an urban setting [45]. Participants will be recruited from within a 16-cell grid (120m^2^/cell) around each park. Thus, the unit of measurement is the individual, but the unit of assignment-to-intervention is neighborhood. We conducted a pilot study in two neighborhoods in 2018 (*n* = 67 participants enrolled) and received funding at the end of the summer in 2019, yielding a truncated field season (*n* = 145 participants). The first full field season for this study will be 2020. To recruit participants, we mail postcards and conduct recruitment activities (e.g., information booths) in each neighborhood. Field staff then visit homes in each study neighborhood to brief potential participants on the study, request participation, and screen for inclusion. We recruit only one English-speaking male or female (≥ 18y) without mobility issues per household, which is at the household’s discretion. To ensure participant comprehension and confirm contact information, we use an electronic text message to finalize enrollment. Participants are then instructed on the correct usage of the accelerometer (Actigraph GT3X), Global Positioning System (GPS) device (Canmore) and cortisol sample collection and instructed that a study shuttle will take them to their scheduled health appointment in our study office. The target total sample is 450 adults to be recruited and retained. To retain participants, we will employ a set of strategies including: 1) sending holiday cards with neighborhood-level results; 2) sending birthday cards; and 3) sending periodic, brief surveys through StANDApp.

### Sample size

Assuming a longitudinal panel design, sample size assessments for the study are based on achieving 82% power to test the hypotheses. The effect measure is a difference-in-differences (DID) — in hypotheses 1–3, i.e., the expected change in PA from baseline to three-years post-restoration in INT parks compared to the corresponding change in CNT parks. Hypotheses 3–6 concern a DID in stress outcomes. A review of the literature on PA [46] and cortisol [47] suggests an effect size (EF) of 0.35 could be posited. Accordingly, we calculate our sample size requirements to detect an EF of 0.35 or better with 82% power. Two other design features are: a serial correlation *r* between repeated measures over time and intra-class correlation (ICC) *ρ* for clustering within neighborhood parks. In community-intervention studies such as ours, *ρ* is small [48–51]. Plausible values from the literature and our own experiences, suggest *r* ≥ 0.35 and *ρ ≤* 0.004. With a total of nine parks (4 INT, 5 CNT), we will need a sample of 450 participants, or 50 per park, to detect EF = 0.35 with 82% power, based on a two-sided test at significance level 5%. Because attrition is expected over the study period, we will recruit a total of 620 participants at t = 0 (baseline) to account for attrition of 15% at t = 1 and another 10% at t = 2 and 5% at t = 3. Our sample size assessment may also be deemed conservative as it does not account for potential influence of fixed covariates, which should increase precision on the intervention effect estimate by reducing residual variance resulting in a positive effect on power.

### Intervention and control parks

Both INT and CNT parks were selected from the same pool of designated ‘Community Open Spaces’ by the City of Detroit Parks & Recreation Department (DPRD). These parks are not maintained as traditional parks and are only mowed once annually. From this pool, we selected CNT parks matched to INT parks based on neighborhood conditions: blighted buildings, greenery, vacant lots, major roads, industrial land use, poverty, and violent/property crime.

#### Intervention description

To combat problems with severe population decline, abandoned and demolished buildings, and huge numbers of unmaintained parks and empty lots [52], DPRD created an Improvement Plan in 2017 to: 1) improve existing parks; 2) strengthen neighborhoods through parks; and 3) convert open spaces into forest buffers, meadows or urban agriculture. DPRD has now implemented this urban rejuvenation effort in partnership with Detroit Audubon.

By restoring unused parks to meadows with native grasses and wildflowers, Detroit Audubon intends to create what it calls *Detroit Bird City*, a city-wide habitat corridor to help conserve hundreds of North American bird species that pass through Michigan using the Detroit River as a migration flyway. Detroit Audubon is scheduled to restore these four parks in 2019–2020. Restorations will include: 1) replacing non-native plant and wildflower species with native species and removing turf and cement; 2) creating trails around each park’s perimeter; 3) adding signage in the park about these efforts; conducting 4) avian and plant biodiversity surveys at each park and 5) guided bird watching walks; and 6) holding community meetings and an annual stewardship event led by Detroit Audubon and DPRD to promote engagement. Each park intervention will be rolled out over a six-month period, including all the activities. Time from restoration to grassland maturation is estimated to be three years. Audubon, in partnership with residents (receiving a paid incentive for maintenance), will keep up these nature areas following restoration.

#### Control description

**Fig. 2:**
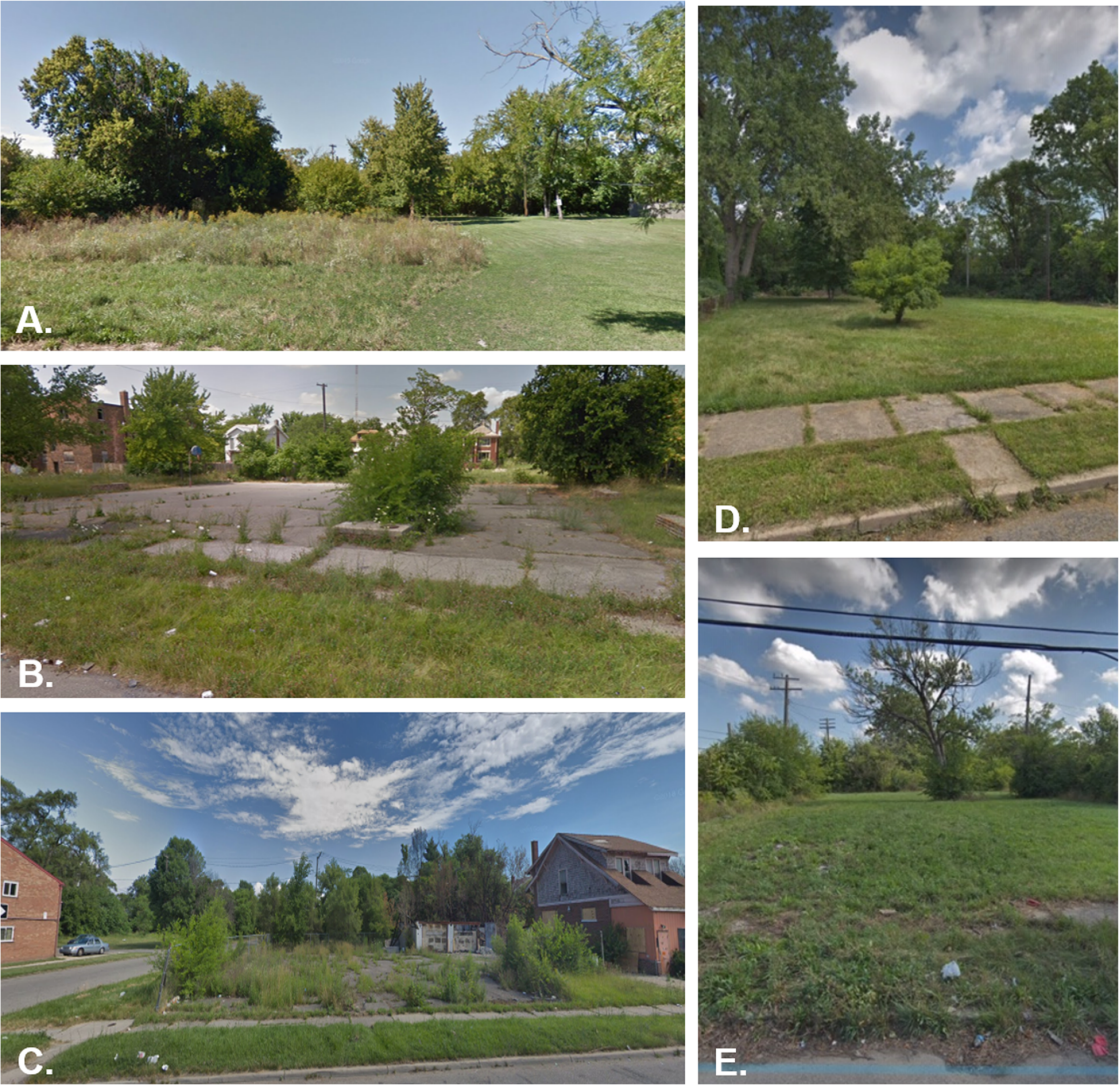
Examples (**a**-**e**) of current conditions in typical participating parks in Detroit, Michigan, U.S.A. Source: Google Street View

In June 2018, we conducted extensive inventories of all nine parks at baseline, evaluating the following: bird species, plant species, and maintenance and usage of parks. Each park had 8–20 bird species, with American Robin and European Starling the most common. Plant diversity was low, with many invasive species and turf grass. Broken cement and signs of dumping were common. Only one park was observed to be used by park-goers (see Fig. 2 for examples of current park conditions). We expect the CNT parks in this project to continue these conditions and to remain unmaintained (only mowed once annually) during the duration of the study.

#### Risk of bias assessment

This study employs the Risk Of Bias In Non-randomized Studies of Interventions (ROBINS-I) tool [53] to assess risk of bias for the pre-intervention components. Specifically, we aimed to reduce bias due to confounding by: i) including a suite of potential individual-level confounders in our survey instrument (detailed below); ii) matching INT and CNT neighborhoods based on baseline area-level factors which may influence both the outcomes and the exposures of interest (detailed above); and iii) consideration of potential co-interventions that might occur (that are related to receiving the intervention and the outcomes). The potential for the intervention to lead to gentrification was identified. Specific items in the surveys will measure perceptions of gentrification and median home price in each study neighborhood will be quantified using publically available data from Redfin, a national real estate brokerage, for each time point. At the time of the intervention, bias in the classification of the intervention will be minimized by collecting objective data on: park usage, plant species, bird species, greenness and signs of care. Post-intervention bias will be assessed at the conclusion of the study.

The participants and the study staff collecting outcome data will be blinded to the condition assignment. This will be accomplished by referring to neighborhoods using a three-letter code and restriction of information about the intervention locations. We will assess blinding at the end of each field season by asking staff which neighborhoods they believe are receiving the intervention. Success will be determined as either i) a majority of “don’t know” responses; or ii) a balance between correct and incorrect responses. Because this is a natural experiment and the researchers have no control over whether the intervention is carried out completely in all locations and to what degree the intervention is discussed throughout the neighborhoods, assessment of blinding of participants is not appropriate.

#### Measurement protocol

All items to be assessed are in Table 1 and include both individual and area-level measures. At recruitment, accelerometers (Actigraph GT3X) and GPS devices (Canmore) on an elastic belt and saliva collection kits will be distributed, and data will be collected for the following week. For subsequent time points, participants will be phoned to arrange an equipment delivery time and schedule a health appointment. Participants will be reminded via StANDApp (detailed below) and/or text to wear the elastic belt and to collect saliva samples. Participants will also take a paper survey at home. Participants will return the equipment, survey and saliva kits at the health appointment or, if they forget an item, will schedule a time to return the item(s) in order to receive the compensation voucher.

**Table 1:** Tools, scale of measurement, and measurements that will be obtained annually 2019–2024. Individual-level refers to measurements collected from individual household participants and area-level refers to measurements collected within and at some points around a park

Tool/Variable	Individual or area-level	Measures obtained
Survey	Individual	Demographics, perceptions of neighborhoods, disease and medication history, diet, perceived stress score, anxiety and depression symptoms, nature-relatedness, perceived safety, acute illness
Physical activity (PA)	Individual	Total activity counts, Moderate-to-vigorous PA, when combined with GPS – green PA
GPS	Individual	Time spent in park, time spent in view of park, when combined with PA – green PA
Cortisol samples	Individual	Total concentration, slope of cortisol from waking to evening
Dried blood spots	Individual	C-reactive protein
Finger-stick A1C	Individual	Hemoglobin A1C levels
Plant surveys	Area	Plant diversity of park, invasive species versus native species
SOPARC	Area	Usage of park
Signs of care	Area	Maintenance level of park
Bird surveys	Area	Bird diversity of park
360 imagery	Area	Greenness of park, negative visual exposures, signs of disorder
AudioMoth acoustic recorders	Area	Bird species, insects, sound diversity, noise
Weather station	Area	Daily precipitation, high temperature

At the health appointment, field staff will collect anthropometrics and dried blood spot (DBS) samples, measure A1C using test strips, and retrieve equipment and cortisol samples. All health data will be entered and then shared with each participant via a health results sheet, which includes resources for free or low-cost healthcare in Detroit.

### StANDApp smartphone app

In partnership with MEI Research Ltd., we have developed a secure mobile app for all study communication, scheduling, and retaining survey deployment. MEI’s PiLR [54] system will allow for the deployment of surveys as a form of monthly contact from the study team to increase retention, sharing health results with each participant, and reminders about health appointments, charging units and wearing belts and taking cortisol samples. Based on pilot work, we determined that some participants may be reticent to use this application on their mobile phone. Accordingly, we will use the app on a voluntary basis. All reminders will also be sent by text message, negating the requirement that all participants use the app.

### Physical activity (primary outcome) measured using accelerometry

PA and sedentary behavior are measured using the ActiGraph accelerometer (ActiGraph model GT3X; Pensacola, FL). The ActiGraph is a triaxial accelerometer that measures acceleration in three planes of motion. Accelerometers capture and filter acceleration signals that are digitized and recorded as count values that are stored in investigator-defined intervals. Raw data are stored and may also be used for analyses.

The accelerometers will be worn on an elastic waistband, placed at the right hip, for all waking hours over seven days at each of the study’s four measurement time points (all during the summer or early autumn). Data will be collected in raw mode (30 Hz) and aggregated to 1-min values for analyses. The primary outcome is total PA counts per week, which has been associated with cardio-metabolic outcomes and biomarkers [22]. Analyses will also include number of minutes spent at given PA intensity levels using cut-points of Freedson et al. (≥1952 cpm) for moderate-to-vigorous intensity PA and Matthews et al. (< 100 cpm) for sedentary behavior, with light PA as 100–1951 cpm [55, 56]. We will also explore PA intensity levels using vector magnitude cutpoints [57]. In addition, these measures will be stratified by whether the PA occurred in the study park, in any greenspace, or elsewhere (based on GPS data). This will provide an indication of the proportion of ‘green PA’ from total PA. Periods of 60 min or more of continuous zeroes are considered non-wear times and not included in the calculation of total wear time. To be included, we will require ≥4 days of PA data (wear time ≥ 8–10 h/day) per time point [58]. If high levels of missing data exist, imputation strategies will be utilized. PA data will be collected at the same time of year (summer months only) to account for seasonality.

### Mobility and park contact measured using GPS data

GPS units (Canmore) will be worn for one week, on an elastic belt with an accelerometer. At the end of the week of observation, data will be downloaded. To calculate the amount of time spent in ‘green PA’, these data will be linked with accelerometer data using time. Accelerometer data are then restricted using a threshold for PA and lux (> 400). Speed is calculated and data are further restricted by excluding speeds >25kmph. This results in total green PA. We will also use GPS data to calculate the actual contact time spent within view of the park [59] and usage of park. To calculate time spent within view of park, we will create ‘viewsheds’ around each park to determine all possible viewpoints from which the park can be seen, accounting for obstacles such as buildings, as we have done previously [60, 61]. To calculate usage of park, all GPS points located within 10 m of the park boundaries will be compiled to calculate time spent in park.

### Stress measures (secondary outcome)

We will measure salivary cortisol, perceived stress and anxiety. Stress alters the dynamics of the diurnal rhythm of cortisol secretion, and people who are chronically stressed exhibit lower morning and higher evening concentrations, yielding flattened slopes [19, 20, 47, 62–67]. Samples will be collected using the passive drool method [68] at waking, and 12-h after waking to permit calculation of time point concentrations and the slope of cortisol from waking to evening. The saliva collection kit contains collection supplies, a response sheet and reminder instructions. Participants will be reminded to take and freeze samples at designated times the day before their scheduled health appointment via StANDApp and/or text. Participants will also be reminded to bring kits to their appointment the next day [69, 70]. In the field, samples will be stored in a manual-defrost freezer and shipped on dry ice (once per measurement period) to Northwestern University’s Laboratory for Human Biology Research where they will be thawed, centrifuged and the supernatant aliquoted into replicate samples in smaller tubes and analyzed in duplicate. Low, medium, and high concentration controls will be prepared in at t = 0 for use throughout the study to monitor inter-assay variability. Cortisol will be measured using the Salimetrics ELISA kit as per manufacturer’s instructions [71].

We will also use two stress indicators via the survey, including: 1) Anxiety and Depression, measured via NIH’s Adult PROMIS-29 Profile v2.0 [72, 73]; and 2) the Perceived Stress Scale [74], comprising 10 items (e.g., feeling nervous) measured on Likert-type scale (0 = low, 40 = max stress). The perceived stress scale has been validated in multiple populations, and the face validity and scale content were ranked high with a Kaiser-Meyer-Olkin coefficient of 0.82 [75]. The scale’s internal consistency reliability was good in multiple languages and convergent validity was supported by expected relationships with other mental health measures, including anxiety and depression [76]. The PROMIS-29’s (our measures of anxiety and depressive symptoms) internal consistency for sub-domains has been shown to be high (Cronbach’s α > 0.88), with adequate structural validity for most domains (CFI > 0.95, RMSEA < 0.05) [77].

### Anthropometric and cardio-metabolic measures (downstream outcomes)

Cardio-metabolic outcomes will include body mass index (BMI), waist-to-hip ratio, blood pressure, glycated hemoglobin A1C (A1C) and C-reactive protein (CRP). Height will be measured twice using a stadiometer (SECA Corp). Weight will be measured twice using a scale with bioelectric impedance capability (Tanita TBF-300). Waist and hip measurements will be taken twice with a Gulick tape, according to World Health Organization (WHO) procedures [78] and the waist-to-hip ratio calculated. Systolic and diastolic blood pressures will be measured as markers of cardiovascular disease (CVD) risk using an automatic upper arm monitor (Omron HEM-711DLX), according to American College of Cardiology recommendations [79]. With the patient seated, two blood pressure measurements will be taken at 30 s intervals. The average of each set of measurements will be used for height, weight, waist and hip measurements and blood pressure. BMI will be calculated and expressed as kg/m^2^. A1C [8, 80] will be measured from blood samples collected from finger-tip sticks using portable analyzers and test strips (A1CNow^+^). Participants will be given these results immediately, including normal ranges and recommendations for consulting a physician.

CRP will be measured to indicate chronic inflammation [81–90], associated with obesity, Type 2 diabetes, cancer, and CVD [89, 91–94]. DBS samples will be collected on Whatman 903 Protein Saver Cards [95, 96] from finger sticks following collection of blood for the A1C analyses. DBS serves as the lowest risk, least invasive blood sampling technique [95]. Circulating levels of CRP in young, healthy adults average 0.8 mg/l. Chronic stress induces small changes in the range of 2–5 ng/l necessitating the use of high-sensitivity assays for CRP [90, 97]. Upon collection of DBS, samples will be transferred to Northwestern University’s Laboratory for Human Biology Research where CRP will be eluted from the DBS and assayed using a CRP assay previously validated in their laboratory for use with DBS [84, 97]. To reduce inter-assay variability and improve quality control, all samples will be stored with humidity sponges and oxygen absorbents and frozen (− 20 °C) until the final data collection year (2022) and then analyzed concurrently, with no expected degradation.

### Survey

Basic demographic data information (age, sex, ethnicity, employment, household composition, length of residence) will be collected at recruitment or when re-contacted at each timepoint. Then, each participant will complete a self-administered paper survey in the privacy of their own home to assess: income, perceptions of the neighborhood [98–102], disease and prescription medication history [103–106], diet [107, 108], perceived stress [74], anxiety and depression symptoms [72, 73] (discussed previously), nature-relatedness [109]; all which were considered potential correlates of device-based PA and stress. Questions about the perceptions of the social and environmental features of neighborhoods have been shown to have moderate to high agreement (rho range = 0.42–0.91) [99]. Family Life, Activity, Sun, Health, and Eating (FLASHE) questions related to diet were reviewed by the scientific experts for consistency with existing, validated measures [108]. The nature-relatedness scale (NR-6) has been shown to demonstrate good internal consistency, temporal stability, and predict happiness, environmental concern, and nature contact [109]. Measures that may serve as confounders include: 1) symptoms of acute illness or infection or prescription medication which could influence biomarker measures; 2) attitudes toward nature using the NR-6, because these may influence behaviors; and 3) perceived safety which may influence stress [110], PA [111, 112], and park usage [113].

### Park observations and imagery

The positive visual exposures of interest include the presence of people using the park, signage, mowing and other signs of care [114], diversity of plants and birds and greenness. Negative visual exposures include the presence of litter, arson, graffiti and broken windows of buildings along the park perimeter (called signs of disorder). We will employ two methods to obtain these data. First, Audubon volunteers will inventory plants in quadrants and count bird species using point counts less than three hours after sunrise, measuring species abundances [115, 116]. Volunteers will be trained by Audubon, and inter-observer reliability assessed by having two or more observers collect data simultaneously but independently [117]. Plant/bird species richness and biodiversity measurement will employ the well-established Shannon Diversity and Simpson Indexes [118, 119] and Evar Evenness Index [116]. Trained graduate students will also make observations of signs of care and the System for Observing Play and Recreation in Communities (SOPARC) [120]. SOPARC provides an assessment of park users’ PA levels, gender, activity types, and estimated age and ethnicity groupings as well as information on a park’s level of accessibility, usability, supervision, and organization with a high internal correlation between items (r = 0.75) [120], and exhibiting high inter-rater reliability (0.80–0.99) [117]. All nine parks, each of which consists of only one target area due their small size, will be observed twice over one day (9 am-2 pm) during randomly scheduled days without rain in summer months, by two raters. INT and CNT parks will be observed at the same time on different days for parallel observations. Signs of care involve observations (presence/absence) of manmade and natural items in a park and provides a categorical quality rating for each item, which is based on qualitative work in Detroit [114]. Second, we will measure visual greenness and negative visual exposures (signs of disorder) via 360^°^ images captured along each park perimeter, using a mounted camera (Samsung Gear 360). Images will be used to measure visual greenness, by quantifying pixels of greenery using methods we developed [121]. Images will also be used to assess negative exposures using Marco et al.’s neighbor disorder coding protocol [122], which has shown acceptable ICCs (0.41–0.60) for the mean level of subscales of physical disorder and decay and has also been validated (showing high correlations) with physical audits, police impressions, and neighborhood socioeconomic status. This virtual auditing approach has been validated across several samples [123, 124].

We will employ two weighting schemes to create individual-level visual exposures from the park measures. First, we will weight each exposure by the actual contact time spent within view of the park in the one-week observation time using the GPS data [59], as described previously. Second, we will calculate the percentage of the viewshed occupied by the park, as seen from the participant’s home by capturing a 360° image from the front door of the home location. We will then create weights using these measures, to be applied to each of the visual exposures above.

### Neighborhood soundscapes

Positive and negative auditory exposures will be assessed by acoustic recordings collected by 90 AudioMoth v1.1.0 acoustic loggers (Open Acoustic Devices). Nine devices will be placed at least 100 m apart randomly in a 500 m grid around each park, and one device will be placed inside each park. We will record for one week in June, corresponding with Audubon’s bird surveys, selecting days to standardize weather conditions. To predict negative auditory exposure, we will calculate environmental noise metrics known to relate to human annoyance, health, and perception including: L_Aeq,T_ (A-weighted equivalent sound level (L) over time); L_Amax_ (maximum A-weighted equivalent L over time); L_A10_ and L_A90_ (A-weighted L exceeded 10 and 90% of time); L_A10_ - LA_90_, LDN (Day-Night average L, where night events receive a 10 dB penalty); L_DEN_ (Day-Evening-High equivalency L); roughness (temporal variation in amplitude), and sharpness [125–128].

To examine positive auditory exposure, trained staff will count the number and duration of sound categories using observations of spectrograms in Raven Pro software (Cornell University, Ithaca, NY). Sound categories are ‘anthropogenic’ (e.g., vehicle, people), ‘geological’ (e.g., wind), and ‘biological’ (e.g., birdsong). Additionally, we will further categorize bird song to species, in order to generate species richness and diversity of the acoustic community [129]. We will combine the duration/frequency and richness of biological with geological sounds into a metric of positive exposure [130]. Using values of each acoustic exposure metric, we will then predict levels throughout the study area using spatial kriging models [131]. We will extract the positive and negative exposure levels for participants at each time point.

### Weather data

While we restrict data collection to summer months (May–September), weather conditions at the time of data collection and the two weeks prior to data collection may influence both exposures of interest and outcomes. Thus, we will compile daily precipitation and high temperature data for every day during data collection and the two weeks preceding data collection for each time point, from the weather station at Detroit airport (DTW) [132, 133].

### Statistical analysis

Assuming a longitudinal panel design, we will compare INT to CNT at baseline using as appropriate ANOVA-F-tests, chi-square tests and non-parametric tests to determine equivalence of potentially confounding physiological and contextual participant characteristics. If substantive differences are found, they will be controlled for in subsequent analyses by regression techniques, guided by the degree of dissimilarity [134]. Equivalence between groups will be assessed on age, sex, ethnicity, employment status, length of residence, marital status, attitudes towards nature, perceived safety, and pre-existing health conditions. We will calculate descriptive statistics for all variables at baseline and each post-restoration time point. Likewise, park characteristics will be summarized for the INT and CNT parks for each time point.

We adopt a regression-based approach to multivariable modeling that addresses features of clustering of participants within parks and correlation over time in repeated assessments. Repeated measures ANOVA, or more apropos, generalized linear (mixed) models (GLMM) will be used [135–137]. Denote by ${\mathit{\mathsf{Y}}}_{\mathit{\mathsf{it}}}$ an outcome in the *i*-th participant in the *h-*th park assessed at time *t*. It is assumed that an individual is primarily exposed to one park. Generally, outcomes are assessed at baseline and at least 3 additional time-points, denoted t = 0, 1, 2, 3. Our hypotheses concern the expected response: ${\textsf{g}}\left({\mu}_{{\textsf{hi}\textsf{t}}}\right)={\mathbf{\textsf{x}}}_{{\textsf{it}}}^{\prime}\beta +\mathbf{\textsf{z}}{\hbox{'}}_{{\textsf{ht}}}{\mathbf{\textsf{b}}}_{{\textsf{hi}}}$ .The GLMM is expressed as ${\textsf{g}}\left({\mu}_{{\textsf{hi}\textsf{t}}}\right)={\mathbf{\textsf{x}}}_{{\textsf{it}}}^{\prime}\beta +\mathbf{\textsf{z}}{\hbox{'}}_{{\textsf{ht}}}{\mathbf{\textsf{b}}}_{{\textsf{hi}}}$ with link function *g* appropriate to type of outcome (continuous, categorical, count, ordinal) random effects ${\mathbf{\textsf{b}}}_{{\textsf{hi}}}$ to capture serial correlation within participant measures and clustering [138]. The covariates ${\mathbf{\textsf{x}}}_{{\textsf{hit}}}$ are participant characteristics, some of which are time-invariant such as the aforementioned sociodemographic variables; ${\mathbf{\textsf{z}}}_{{\textsf{ht}}}$ are characteristics of park *h* at time *t.* Minimally, the predictor variables are: a single indicator for GROUP, with CNT as referent, three indicators for TIME, corresponding to t = 1, 2, 3 with baseline t = 0 as referent, and GROUP×TIME interactions. A measure of exposure to environmental stimuli is included in participant characteristics. The GLMM allows us to formulate and test hypotheses on functions of the regression parameters β, including: i) point-in-time comparison between INT and CNT, e.g., at each follow-up year; ii) time-averaged comparison between INT and CNT; iii) within group comparison for change over time; and iv) change in INT compared to the corresponding change in CNT via DID. SAS Software v9.4 (Analytics 15.1 or higher) will be used for statistical analyses.

To address missingness in our response data, we will employ strategies for imputation [139]. The techniques described above allow missing at random (MAR). Inverse probability weighting [140] will be investigated to accommodate missing data patterns that are not MAR. To minimize bias in analysis, we will adjust for factors that may be unbalanced between groups and examine the robustness of our conclusions under deviations of model assumptions. Below, we outline specific analyses for each hypothesis to be tested.

#### Hypothesis 1

We will separately evaluate average activity counts/minute and average moderate-to-vigorous minutes over the one-week observation period for each of the data collection periods. The predictor of interest is binary: INT versus CNT.

#### Hypothesis 2

The primary outcome, ‘green PA’, will be average activity counts/minute and average moderate-to-vigorous minutes over the one-week observation period while in parks and greenspaces in Detroit, as a subset of all activity.

#### Hypothesis 3

We hypothesize there will be an association between PA and the visual and auditory exposures. Instead of a binary predictor of interest, we will use continuous predictors for visual and auditory park exposures. Interaction terms between positive and negative exposures will also be assessed, as evidence suggests that negative sounds deplete the restorative benefits of natural sounds, [141] and that parks are often dominated by noise [45, 142].

#### Hypothesis 4

Each stress outcome will be evaluated separately and treated continuously. We will also explore potential mediators (e.g., PA) by adding each to mixed models as fixed terms, and adjusted mediation and partial correlation coefficients explored. We will adjust for prescription medication history and symptoms of acute illness as potential confounders.

#### Hypothesis 5

We will assess relationships with each stress indicator, in turn.

#### Hypothesis 6

We hypothesize there will be an association between each stress indicator and these exposures.

#### Hypothesis 7

We will compare INT and CNT groups from baseline through three-years post-restoration for changes in CRP and A1C (continuously measured) between INT and CNT groups. We will also consider the inclusion of prescription medication history and symptoms of acute illness as potential confounders.

#### Hypothesis 8

We will compare INT and CNT groups from baseline through three-years post-restoration for changes in blood pressure, hip-to-waist ratio and BMI (continuously measured) between INT and CNT groups.

Despite our best effort to recruit and retain a longitudinal panel of participants, we may face problems with attrition higher than the projected attrition rates of 15% at t = 1, another 10% at t = 2, and 5% at t = 3. With these levels, our longitudinal panel becomes unbalanced but valid inference can be made under the assumption that attrition is at random (MAR). We describe another strategy, *repeated cross-sections*.

By repeated cross-sections we mean a series of independent samples are drawn at the subsequent times t = 1, 2, 3 following the initial baseline sample at t = 0. It could happen that a participant in the baseline sample is also in any of the subsequent samples, but this is at random. With repeated cross-sectional samples, we cannot estimate *within participant* change in outcomes, which is the key advantage of the longitudinal panel approach. However, we can still assess patterns of change at an aggregate level as explained in the following text.

Consider an outcome ${\mathit{\mathsf{Y}}}_{\mathit{\mathsf{it}}}$ at time *t* in the *i-*th participant, and the linear model ${{\textsf{Y}}}_{{\textsf{i}\textsf{t}}}={\mathbf{\textsf{x}}}_{{\textsf{i}\textsf{t}}}^{\prime}\beta +{\alpha}_{{\textsf{i}}}+{\varepsilon}_{{\textsf{i}\textsf{t}}}$ with exogenous covariates ${\mathbf{\textsf{x}}}_{{\textsf{it}}}$, *t* = 0, 1, 2, 3. We aggregate each sample into *C* cohorts defined as participants who share common characteristics such as age, sex, neighborhood park, etc. Aggregation of participants to the cohort level *c* replaces the model by the cohort model ${\overline{{\textsf{Y}}}}_{{\textsf{c}\textsf{t}}}={\overline{{\mathbf{\textsf{x}}}^{\prime}}}_{{\textsf{c}\textsf{t}}}\beta +{\overline{\alpha}}_{{\textsf{c}}}+{\overline{\varepsilon}}_{{\textsf{c}\textsf{t}}}$, *c* = 1, …,*C*, *t* = 0, 1, 2, 3. (Bar notation refers to averages over participants within cohort/time). Our observed data become a pseudo-panel of repeated observations on cohorts over time, and estimation of differences between INT and CNT cohorts could be compared over time, although the cohorts are not comprised of the same participants from one time point to the other. Some technical issues will need to be addressed to ensure a consistent estimator of *β* [143–146].

## Discussion

At the time this investigation was initiated, to our knowledge no experimental study has examined the visual and auditory exposures to greenspace to illuminate the twinned effects on PA and stress, and downstream cardio-metabolic health. Previous experimental studies have focused on the addition of conventional park equipment and infrastructure, which tend to be very expensive interventions. Additionally, we apply a rigorous measurement protocol that uses device-based/objective measurement of both exposures and outcomes to evaluate the effects of the intervention on PA, stress and cardio-metabolic outcomes. Further, and importantly, this study involves measurement of individual-level variation in positive and negative exposures using geospatial techniques, rather than assuming equal exposure among all nearby residents. A recent report from WHO on the effectiveness of greenspace interventions on health noted that such interventions are promising avenues to improve physical and mental health in cities, particularly in low-income neighborhoods, although evidence is still needed to confirm the effect of urban greenspace interventions in deprived populations. This study addresses several recommendations from the report. The effectiveness of those recommendations is yet to be determined in a manner that will allow researchers and practitioners to provide better evidence-informed policies and practices in low-income urban neighborhoods.

## Data Availability

Not applicable.

## Abbreviations

A1C: Glycated hemoglobin A1C
BMI: Body Mass Index
CNT: Control park neighborhoods in our study
CRP: C-reactive protein
CVD: Cardiovascular disease
DBS: Dried blood spots
DID: Difference-In-Differences
DPRD: City of Detroit Parks and Recreation Department
EF: Effect size
FLASHE: Family Life, Activity, Sun, Health, and Eating
GLMM: Generalized Linear (Mixed) Models
GPS: Global positioning system
ICC: Intra-class correlation
INT: Intervention park neighborhoods in our study
IRB: Institutional Review Board
MAR: Missing at random
NCI: National Cancer Institute
NIH: National Institutes of Health
NR-6: Nature-relatedness, 6 question
PA: Physical activity
ROBINS-I: Risk Of Bias In Non-Randomized Studies of Interventions
SOPARC: System for Observing Play And Recreation in Communities
StAND: Study of Active Neighborhoods in Detroit
WHO: World Health Organization
